# Optimal dynamic regimens with artificial intelligence: The case of temozolomide

**DOI:** 10.1371/journal.pone.0199076

**Published:** 2018-06-26

**Authors:** Nicolas Houy, François Le Grand

**Affiliations:** 1 University of Lyon, Lyon, F-69007, France; CNRS, GATE Lyon Saint-Etienne, F-69130, France; 2 emlyon business school, Écully, F-69130, France; ETH Zurich, Zurich, CH-8092, Switzerland; Universita degli Studi di Catania, ITALY

## Abstract

We determine an optimal protocol for temozolomide using population variability and dynamic optimization techniques inspired by artificial intelligence. We use a Pharmacokinetics/Pharmacodynamics (PK/PD) model based on Faivre and coauthors (Faivre, et al., 2013) for the pharmacokinetics of temozolomide, as well as the pharmacodynamics of its efficacy. For toxicity, which is measured by the nadir of the normalized absolute neutrophil count, we formalize the myelosuppression effect of temozolomide with the physiological model of Panetta and coauthors (Panetta, et al., 2003). We apply the model to a population with variability as given in Panetta and coauthors (Panetta, et al., 2003). Our optimization algorithm is a variant in the class of Monte-Carlo tree search algorithms. We do not impose periodicity constraint on our solution. We set the objective of tumor size minimization while not allowing more severe toxicity levels than the standard Maximum Tolerated Dose (MTD) regimen. The protocol we propose achieves higher efficacy in the sense that –compared to the usual MTD regimen– it divides the tumor size by approximately 7.66 after 336 days –the 95% confidence interval being [7.36–7.97]. The toxicity is similar to MTD. Overall, our protocol, obtained with a very flexible method, gives significant results for the present case of temozolomide and calls for further research mixing operational research or artificial intelligence and clinical research in oncology.

## Introduction

One of the salient features of treatments in oncology is the persistent gap prevailing between standard drug regimens, corresponding to the official recommendation, and the actual drug regimens that are applied at bedside. For instance, Atkinson et al. [4] perform a retrospective study on the drug regimens that have been administered to patients with metastatic renal cell cancer. They conclude that in a significant number of cases, alternative protocols have been administered at bedside due to the patient reaction to the standard protocol. Furthermore, these alternative regimens are found to deliver in some cases more favorable outcomes than the standard protocol. This gap between official recommendations and actual prescriptions calls for improvement in recommended protocol design, in terms of both efficacy and toxicity, as well as for a better consideration of heterogeneity in patient drug responses (see [5] for a review). As advocated by several authors in [6] and [7], computational oncology is a very promising route to optimize the design of drug regimens since, given the large number of protocol possibilities, standard clinical trials are of little help for an exhaustive exploration. In this perspective, Pharmacokinetics/Pharmacodynamics (PK/PD) models, that formalize the trade-off existing between toxicity and efficacy for a given drug, are of particular interest and have for instance shown to be helpful in the clinical design of protocols [8–10]. PK/PD models have already been used to determine optimal protocols in some particular situations. In a first approach (see [11] for a seminal reference or [12, 13] for reviews), these models have been used to determine the optimal drug quantity, while the regimen schedule was considered as given. A second approach consists in partly relaxing the constraint of fixed schedule and in optimizing upon both drug quantities and treatment days. The cycle, *i.e.*, the length and sequence of treatment and rest periods, is still considered to be fixed. For instance, Barbolosi and colleagues [14] consider the administration of vinorelbine, with a cycle of 7 days and a fixed weekly total dose of 150 mg. They prove that the alternative protocol consisting of 60 mg, 30 mg and 60 mg on days 1, 2, and 4 provides a better efficacy, and a similar toxicity, than the standard protocol consisting of the constant amount of 50 mg on days 1, 3, and 5. Furthermore, Meille et al. [15] and Hénin et al. [16] have used optimization results to provide guidance in designing protocols for phase I/II clinical trials.

This paper belongs to this trend and also relies on a PK/PD model to determine optimal chemotherapy regimen. We investigate the case of temozolomide, used in the treatment of some brain cancers, notably for children. Our optimization exercise is innovative along two dimensions. First, we fully relax the schedule constraint. We determine the optimal protocol over a 336-day period, but we do not impose any cycle or weekly pattern. The period length of 336 days corresponds to a multiple of the cycle length of the standard Maximum Tolerated Dose (MTD) protocol for temozolomide. More precisely, in every day of the simulation period, we determine which treatment dose –including no dose– is optimal. Giving up cycles enables us to quantify the possible gains from opting for a fully unconstrained approach. Even though the existence of cycles are often considered as an important feature of clinical trials, we believe that our computational approach is a very good opportunity to assess the benefits of removing cycle constraints. The second innovation is that the optimal protocol is not only designed for a “median” patient, but for an heterogeneous population. Indeed, we take into account the individual patients specificities through heterogeneity in the population pharmacokinetics. We rely on the data of Panetta and coworkers [3], who investigate population pharmacokinetics for temozolomide. Our selected protocol minimizes the tumor size in the population, while limiting the toxicity for the whole population. The tumor size is our proxy for efficacy and we measure toxicity by the minimal normalized absolute neutrophil count (ANC) over the protocol period.

In our *in-silico* experiments, our optimized protocol yields unambiguously promising results. We compare our results to the standard MTD protocol, which corresponds to the administration of 200 mg/m^2^ from day 1 to day 5 for a total cycle of 28 days. Our optimal treatment yields a tumor size on average 7.66 times smaller than with the MTD protocol –the 95% confidence interval for the size factor being [7.36–7.97]. This smaller tumor size on average is accompanied by a reduction in efficacy dispersion. The 95th percentile of the tumor mass distribution amounts to 111.4 grams with MTD and only 33.6 grams with our optimal protocol. This better efficacy in terms of average and dispersion does not come at the cost of a greater toxicity. Indeed, a smaller share of the population experiences a normalized ANC nadir below the acceptable threshold when our optimal protocol is administered. We set the acceptability threshold for normalized ANC nadir to 2.7%, which corresponds to the 5th percentile of the normalized ANC nadir for a population to which the MTD protocol has been administered. With our protocol, only 1.78% of the population experiences a normalized ANC nadir below the acceptability threshold, while by definition this proportion amounts to 5% with the MTD protocol. For a reference point, note that a typical ANC value is 7000 cells/mm^3^ to 8000 cells/mm^3^ [17]. [18] defines that an ANC of 1500 cells/mm^3^ should be considered to be abnormally low and severe infections occur at values below 500 cells/mm^3^. [19] define a neutropenia related event as an ANC nadir below 250 cells/mm^3^.

What does our optimal protocol look like? First, our protocol exhibits a pseudo-periodicity. Every 5 weeks approximately, the protocol features several consecutive days of treatment –typically, three to five. Each of these periods of consecutive treatment days is followed by a period lasting approximately 4 weeks, during which few –three to five– treatment days take place. Even though we do not impose *a priori* any periodicity or any cycle, the optimal protocol features some sort of regularity, which makes it look like a distant cousin of MTD. The two main differences is that treatment period is not always exactly five days and there is no such a thing as a 23-day rest period. Treatment days always occur between two periods of several consecutive treatment days. Our optimal protocol can therefore be seen as an hybrid between the standard MTD and metronomic chemotherapy protocols –which loosely speaking involve the administration of low doses with no prolonged break.

Even *in-silico*, determining the optimal treatment is not an easy task. Since we relax the periodicity constraint, treatments can occur at any day. Because our simulation period covers 336 days, the number of possible protocols is vertiginous. Indeed, if only allowing for two possibilities per day (treatment or no treatment), the number of possible protocols over the period amounts to 2^336^ which has an order of magnitude 10^101^. If we assume that computing the efficacy and toxicity for one protocol and for one patient –and remember that we will deal with patient’s heterogeneity, so that we will compute average performance over entire populations– requires one second, testing for all protocol possibilities implies a computational time in the order of magnitude of 10^94^ years. For the sake of comparison, the solar system is approximately 4.6 × 10^9^-year old. Even if we could massively parallelize the computations, the result would remain far out of reach at a human scale. In practical terms, this means that relying on standard optimization techniques, such as dynamic programming, is not a feasible option to determine optimal protocols, given current computational power. Interestingly, *in-silico* experiments, in a first brute-force approach, are not of a greater help than clinical trials to design optimal protocols, even though they are simpler and cheaper to implement. *In-silico* experiments must therefore be accompanied by high-performance optimization heuristics that enable to come up with a close-to-optimal solution in a reasonable time frame. The heuristics we rely on in this article borrows from the field of artificial intelligence and in particular from the class of the so-called Monte-Carlo tree search algorithms (see [20] for a seminal reference). This class of algorithms has initially be designed for two-player games. A famous application is the program AlphaGo, that has defeated a number of Go champions –see [21] for a description. We have modified and adapted Monte-Carlo tree search algorithms to handle optimization problems in presence of uncertainty. This enables us to circumvent the curse of dimensionality and to determine the (close to) optimal protocol for temozolomide administration in a reasonable amount of time, while taking into account population variability in pharmacokinetics. As shown by our results on toxicity and efficacy, such optimizing heuristics are very complementary to PK/PD models and offer a promising route for designing optimal protocols in oncology.

## Materials and methods

### PK/PD model and simulations

The PK/PD model of temozolomide we rely on borrows from two sources. First, the pharmacokinetics of temozolomide, and the pharmacodynamics of efficacy come from Faivre and colleagues [1]. Second, for the pharmacodynamics of toxicity, we use the model of Panetta and coworkers [2].

We now provide a brief description of the PK/PD model. First, pharmacokinetics follows the original paper of Panetta et al. [3] and relies on a standard one-compartment model. We use a population model for pharmacokinetics, implying that pharmacokinetic parameters –that drive the temozolomide absorption and plasmatic concentration– are individual-dependent. Second, the pharmacodynamics for efficacy is implemented by an interface model, *à la* Meille et al. [22]. The principle of this two-interface model is that temozolomide affects both endothelial and cancer cells, but that the latter are more sensitive to temozolomide than the former. The tumor mass is assumed to follow a Gompertz model in absence of treatment. The calibration is such that the tumor mass doubles within 40 days in absence of treatment. Temozolomide is assumed to impede tumor growth through two channels. First, standard cytotoxic effects on cancer cells diminish tumor size, but these effects are dampened down by drug resistance due to repeated exposure. Second, anti-angiogenic effects, which come from the killing of endothelial cells, contribute to limit tumor growth. Contrary to cancer cells, endothelial cells do not exhibit any drug resistance in the model.

Finally, the pharmacodynamics of toxicity relies on a physiological model of hematopoiesis, describing the myelosuppressive effect of temozolomide. The model was originally proposed by Panetta and coworkers [2]. A physiological model is needed because approximating the toxicity measure by the area under the curve (AUC) of temozolomide plasmatic concentration, even though partly successful [23], is found to actually be a very imperfect measure of the actual toxicity (see [2]). The physiological model of hematopoiesis relies on a three-compartment model that accounts for the successive development stages of proliferating cells in the bone marrow. Starting as pluripotential stem cells, they progressively mature into differentiated blood cells (platelets, red blood cells, and white blood cells). The granulocyte colony stimulating factor (G-CSF) affects the growth of proliferating cells through a negative feedback effect. Regarding toxicity, temozolomide acts as an on/off switch on the growth of proliferating cells in the bone marrow. More precisely, whenever the plasmatic concentration of temozolomide crosses a given threshold, the growth of proliferating cells is completely shut down, which ultimately harms neutrophil counts. S1 Appendix contains the full-fledged mathematical formulation of the model, as well as the parameter calibration we use. A detailed numerical analysis of the model can be found in [24], where the properties of the model are discussed in regards to the medical literature.

We simulate the PK/PD model over a time length of 336 days, which corresponds to 12 full cycles of the standard MTD protocol. All computations are implemented in C++. For each protocol, we assess its efficacy and toxicity for a given patient as follows.

- *Efficacy:* the logarithm of the tumor size (in grams) at the final day of the 336-day period. A high efficacy corresponds to a small tumor size.
- *Toxicity:* the normalized ANC nadir, *i.e.*, minimal normalized ANC (in %) obtained over the simulation period of 336 days. A high toxicity means a small normalized ANC nadir. Note that the algorithm is flexible enough to handle multidimensional measures of toxicity and for instance to also include the time length below a given ANC threshold, as well as the minimal ANC value before a new treatment sequence (as done in [15, 16]). We have chosen to focus on the ANC nadir only, which is unambiguously considered as the relevant measure of toxicity (see the full prescribing information [25] about Temodar^®^ capsules, which is the original brand name of temozolomide).

Since we use a population model for the pharmacokinetics, the drug absorption is not constant throughout the population and consequently, plasmatic concentration of temozolomide for a given protocol also varies across patients. Therefore, even though the pharmacodynamics for both toxicity and efficacy is constant in the population, the actual efficacy and toxicity of a given protocol, that depend on the drug plasmatic concentration, vary across patients. A given protocol is consequently not characterized by a unique pair of efficacy and toxicity, but by a population distribution of efficacy and toxicity values.

**Fig 1:**
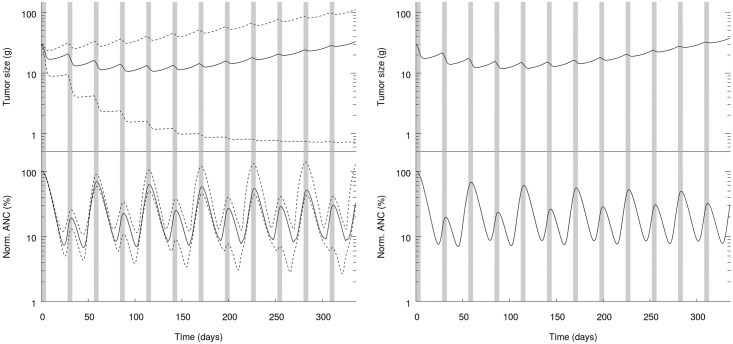
Tumor size (top) and normalized ANC (bottom) as a function of time. Grey areas are treatment periods. Panel A: Population variability. Solid line: median, dashed lines: 5th and 95th percentiles. Panel B: No variability.

We illustrate these aspects in panel A of Fig 1, where we show the evolution over time of the normalized ANC and the tumor size for MTD, taking into account variability in population pharmacokinetics. Grey areas correspond to treatment periods. In panel B of Fig 1, we similarly show the normalized ANC and the tumor size in absence of variability, *i.e.* with parameters set to the average values of the population distribution.

From Fig 1, we observe that the impact of population variability on both efficacy and toxicity is very sizable. The tumor size at the final date varies from 0.7 gram to 111.4 grams for the 5th and 95th percentiles, while the median amounts to 33.0 grams. As for efficacy, the variability in toxicity is also significant. The normalized ANC nadir for both percentiles are 2.7% and 10.7% respectively, while the median value is 6.7%. Finally, the comparison of both panels of Fig 1 highlights that the pharmacodynamics of the median patient (panel B in Fig 1) is quantitatively very similar to the median pharmacodynamics of the population (panel A in Fig 1).

### Optimization algorithm

We provide a detailed version of the pseudo-code in Algorithm 1. All statements following the sign ‘//’ are comments. The algorithm relies on the PK/PD model for temozolomide described above, that we do not make explicit here for the sake of conciseness. In the algorithm, a patient is characterized by a set of particular values for pharmacokinetics parameters –that are fixed over time– and a pair of efficacy and toxicity values that evolves over time, reflecting the administered protocol and the dynamics imposed by the PK/PD model. A population is a collection of patients and is characterized at every day by the distribution of efficacy and toxicity values.

The algorithm consists of two main parts. The first part is the procedure PkPd, which is an auxiliary module for the function dealing with the dynamic system. More precisely, the procedure PkPd(*Pop*, *NbDays*, *prot*) updates the efficacy and toxicity for all patients of the population *Pop*. At the current date, this population is characterized by a collection of PK/PD values, such as the tumor size and the normalized ANC. We update these values by running the PK/PD model (described above and whose exact formulation can be found in S1 Appendix) applied to every patient of the population *Pop* for *NbDays* days, starting at the current date. In these simulations, we assume that the protocol *prot* is administered to every patient. Note that we denote by *⌀* the protocol with no treatment dose. The second part is the core of the algorithm. Function OptimProtocol (*P*, *Horizon*, *nadir*_min_, *θ*, *N*_*MC*_) computes the optimal protocol for the patient population *P*, over the horizon *Horizon*. In most of our simulations, the horizon is 336 days, while the population counts 360 patients randomly drawn from population pharmacokinetics (we compare results after 336 days, but nothing in our algorithm implies a border effect at 336 days). The role of parameters *nadir*_min_, *θ*, and *N*_*MC*_ will be made explicit below.

The core of the function is to determine, at a given day *d* of the simulation, which drug dose, including no dose, is optimal. In all generality, we should test for a large number of possible doses, between no dose (*i.e.*, 0 mg/m^2^) and the maximum tolerated dose, which is 200 mg/m^2^ per day for temozolomide. However, we can take advantage of the PK/PD model for temozolomide to simplify the dosing possibilities. Indeed, as shown in [24], it is always optimal, in the case of the PK/PD model under consideration, to administer a dose very close to the maximum tolerated dose of 200 mg/m^2^. Medium or low temozolomide doses trigger the same toxicity as large doses, while their efficacy is much lower. This is mainly due to three properties of the temozolomide PK/PD model we rely on. First, the toxicity of temozolomide is modeled as shutting-down the production of proliferating cells in the bone marrow. This shut-down is binary (either full production or no production) and is triggered by a relatively low plasmatic concentration of temozolomide. Consequently, small dose administrations have a similar toxicity effect than large doses. Second, the major efficacy channels are also binary and become effective only at large plasmatic drug concentrations. Therefore, small drug doses have barely no impact on efficacy. Third, the plasmatic clearing of temozolomide is relatively fast. Large drug doses therefore do not have long lasting effects. In consequence, as can be seen in OptimProtocol, we can reduce our investigation of possible doses to a binary choice between a 200 mg/m^2^ dose and no dose. Note that the no-dose case corresponds to an optimal choice but does not embed the possibility of a patient skipping one day of the treatment. Modeling missing treatment possibilities would require a specific probabilistic modeling, reflecting patients’ skipping behavior. Our algorithm is flexible enough to be able to take into account these behaviors. However, we believe that this extension is of interest on its own and we leave it for future research.

**Algorithm 1** Optimizing drug administration—Algorithm H.

**Require:** A PK/PD model with population data for pharmacokinetics.

 1: **procedure** PkPd(*Pop*, *NbDays*, *prot*)

  // Update the characteristics of the population *Pop* by simulating the PK/PD model for *NbDays* days, and assuming that the protocol *prot* is administered to every patient.

 2:   **for** *p* ∈ *Pop* **do**

 3:    **Simulate** PK/PD model for patient *p* and protocol *prot*

 4:   **end for**

 5: **end procedure**

 6: **function** OptimProtocol(*P*, *Horizon*, *nadir*_min_, *θ*, *N*_*MC*_)

   // Determine the optimal protocol for the patient population *P*, for a length horizon equal to *Horizon*

  // *nadir*_min_: min. acceptable norm. ANC nadir; *θ*: max. population share with a norm. ANC nadir below *nadir*_min_

                          // *N*_*MC*_: horizon in simulations for fictive populations

 7:   OptiP ← empty vector of length *Horizon*

 8:   **for** *d* ← 1 to *Horizon* **do**

 9:    **for** *i* ← 1 to 4 **do**

 10:     *P*_*i*_ ← Copy of population *P*

 11:    **end for**

 12:    **Administer** a 200 mg/m^2^ dose to populations *P*_1_ and *P*_3_ at day *d*

 13:    **for** *i* ← 1 to 2 **do**

 14:     **Call** PkPd(*P*_*i*_, *N*_*MC*_, ⌀)

 15:     **Call** PkPd(*P*_*i*+2_, *N*_*MC*_, MTD protocol)

 16:    **end for**

 17:    **for** *i* ← 1 to 4 **do**

 18:     %*Tox*_*i*_ ← Share of *P*_*i*_ with normalized ANC nadir ≤ *nadir*_min_

 19:     **if** %*Tox*_*i*_ ≥ *θ* **then**

 20:      *Eff_i_* ← ∞

 21:     **else**

 22:      *Eff_i_* ← Average of the log tumor size for *P*_*i*_ at day *d* + *N*_*MC*_

 23:     **end if**

 24:    **end for**

 25:    *i*_min_ ← argmin{*Eff*_1_, *Eff*_2_, *Eff*_3_, *Eff*_4_}

 26:    **if** *i*_min_ ∈ {1, 3} **then**

 27:     **Call** PkPd(*P*, 1, 200mg/m^2^)    // Administration of a 200 mg/m^2^ dose at day *d*

 28:     OptiP(*d*) ← 1

 29:    **else**

 30:     **Call** PkPd(*P*, 1, ⌀)         // No dose administration at day *d*

 31:     OptiP(*d*) ← 0

 32:    **end if**

 33:   **end for**

 34:   **Return** OptiT

 35: **end function**

In order to determine which of the 200 mg/m^2^ or no dose is optimal, function OptimProtocol compares the future toxicity and efficacy outcomes of the two dosing possibilities. If the 200 mg/m^2^ dose offers a better average efficacy than no dose, while exposing the population to an acceptable toxicity level, then the recommended action for day *d* will be the administration of a 200 mg/m^2^ dose. Conversely, if the no dose yields a better efficacy or if the toxicity with the 200 mg/m^2^ dose is too high, then the recommended action is no dose for day *d*. The function OptimProtocol therefore returns the optimal protocol ‘OptiP’, which is a vector of length 336, containing only 0 and 1, where 1 refers to a 200 mg/m^2^ dose and 0 to no dose.

The issue in the previous operation is that assessing future outcomes for toxicity and efficacy relies on future dose administrations that are unknown by construction. We therefore need to make assumptions. We will suppose that the assessment of future outcomes relies on future protocols that are *a priori* fixed and are set to either the no treatment protocol or MTD. More precisely, this assumption is used as follows. The function OptimProtocol compares the efficacy and toxicity of four different fictive populations. Each of these fictive populations is a copy of the population *P* at day *d* and they differ from each other by the initial dose (0 or 200 mg/m^2^) and by the continuation protocol (no treatment protocol or MTD). Then, the toxicity and efficacy for these four populations are updated and computed *N*_*MC*_ days later –at day *d* + *N*_*MC*_. Next, we select the population featuring the best efficacy for an acceptable toxicity level. The best efficacy corresponds to the smallest average log tumor mass after *N*_*MC*_ days. Our efficacy objective is indeed expressed in logarithm of the tumor size. With such a non-linear objective function, a 10 gram decrease has a greater weight for an initial tumor mass of 20 grams than for an initial mass of 80 grams. The toxicity will be considered to be acceptable if less than a proportion *θ* of the population experiences a normalized ANC nadir below *nadir*_min_. The proportion *θ* is simply computed as the number of patients in a given population whose normalized ANC nadir is below the threshold, divided by the total size of the population. The parameter *nadir*_min_ is therefore our acceptability threshold for normalized ANC nadirs. In our simulations, we set *N*_*MC*_ = 40 days. This value may seem small but increasing it further has a negligible quantitative impact on results. We also set *nadir*_min_ = 2.69%, which corresponds to the 5th percentile of normalized ANC nadirs for a population to which MTD has been administered. Finally, we calibrate *θ* to 2%, which guarantees that the toxicity in the actual population *P* remains acceptable. We provide a sensitivity analysis to the calibration of the parameter *θ* in S2 and S3 Appendices. Sensitivity results are consistent with intuition and confirm our findings.

Finally, the dose administered at day *d* will be determined by the selected fictive population. If the selected population did receive an initial dose (no matter the continuation protocol), the administered dose to population *P* for day *d* is 200 mg/m^2^. Conversely, if the selected population did not receive any initial dose, population *P* is not administered any temozolomide at day *d*. The population *P* is then updated until day *d* + 1. The process repeats until the end of the 336-day horizon is reached.

In the remainder, we will refer to this optimal protocol as the heuristic –or H– protocol.

## Results

### Absence of variability

As a benchmark, we implement our optimization algorithm in absence of variability. The pharmacokinetics is identical for all patients, as in panel B of Fig 1 for the administration of the MTD protocol. In that case, we can readily apply Algorithm 1. However, since there is no variability, parameters need to be slightly modified. First, the population size is reduced to 1, since the pharmacokinetics for all patients is the same. Second, we correspondingly need to set *θ* = 1 since we treat a unit population and we want this unique patient not to experience a normalized ANC nadir below the acceptable threshold *nadir*_min_. Also, we consider this toxicity acceptable threshold to be the toxicity implied by the MTD protocol (7.00%).

Our results are summarized in Table 1. Compared to MTD, the H protocol features a similar toxicity level by construction, but a much smaller tumor mass. With the H protocol, the tumor mass is divided by almost 6 compared to MTD and reaches the value of 6.51 grams, compared to 38.15 grams for MTD.

**Table 1:** Protocols comparison in absence of variability.

Protocol	Norm. ANC nadir (%)	Tumor mass (g)
MTD	7.00	38.15
H protocol	7.00	6.51

### Pharmacokinetic variability

We now turn to the output of the H protocol in presence of pharmacokinetics variability across patients. So as to observe its efficacy and toxicity, the H protocol is administered to a population of 3,200 patients drawn from the pharmacokinetics distribution. We compare the H protocol to MTD, administered to the same population of 3,200 patients. We summarize our results in Table 2.

**Table 2:** Protocols comparison with pharmacokinetics variability. Median values and in square brackets, the 5th and 95th percentiles.

Protocol	Norm. ANC nadir (%)	Tumor mass (g)
MTD	6.74 [2.67 − 10.76]	32.99 [0.72 − 111.40]
H protocol	4.17 [2.74 − 6.22]	1.80 [0.60 − 33.55]

Our results are unambiguous. The H protocol delivers a much better efficacy than MTD. The median tumor mass is 1.80 grams compared to 32.99 grams with MTD. The differences though still impressive, are slightly smaller for the 5th and 95th percentile. On average, the H protocol yields a tumor mass approximately 7.66 times smaller than MTD! The 95% confidence interval for the size factor is [7.36–7.97]. Furthermore, this smaller average value comes with a smaller dispersion of the tumor mass across patients. While with MTD the range of tumor masses between the 5th and 95th percentiles varies from 0.72 gram to 111.40 grams, the same range with the H protocol only covers the interval between 0.60 gram and 33.55 grams. In other words, the H protocol offers a better efficacy in terms of average *and* of dispersion.

This better efficacy does not come at the cost of greater toxicity. Indeed, the population share experiencing a toxicity below the acceptability threshold is smaller with the H protocol than with MTD. More precisely, the 5th percentile of toxicity with the H protocol corresponds to a normalized ANC nadir equal to 2.74%, which is very close to –and slightly above– the 5th percentile in the MTD case. However, we can observe that, with no impact on our objective measure, the dispersion of the normalized ANC nadir in the population with H protocol is much smaller. Indeed, with the H protocol, 95% of the population experiences a normalized ANC nadir below 6.2%, while with MTD this 95th percentile reaches 10.76%. Population toxicity is therefore more concentrated around the acceptability threshold with the H protocol than with MTD. This better control of toxicity with the H protocol can be an important factor in explaining its better efficacy in terms of average and dispersion.

**Fig 2:**
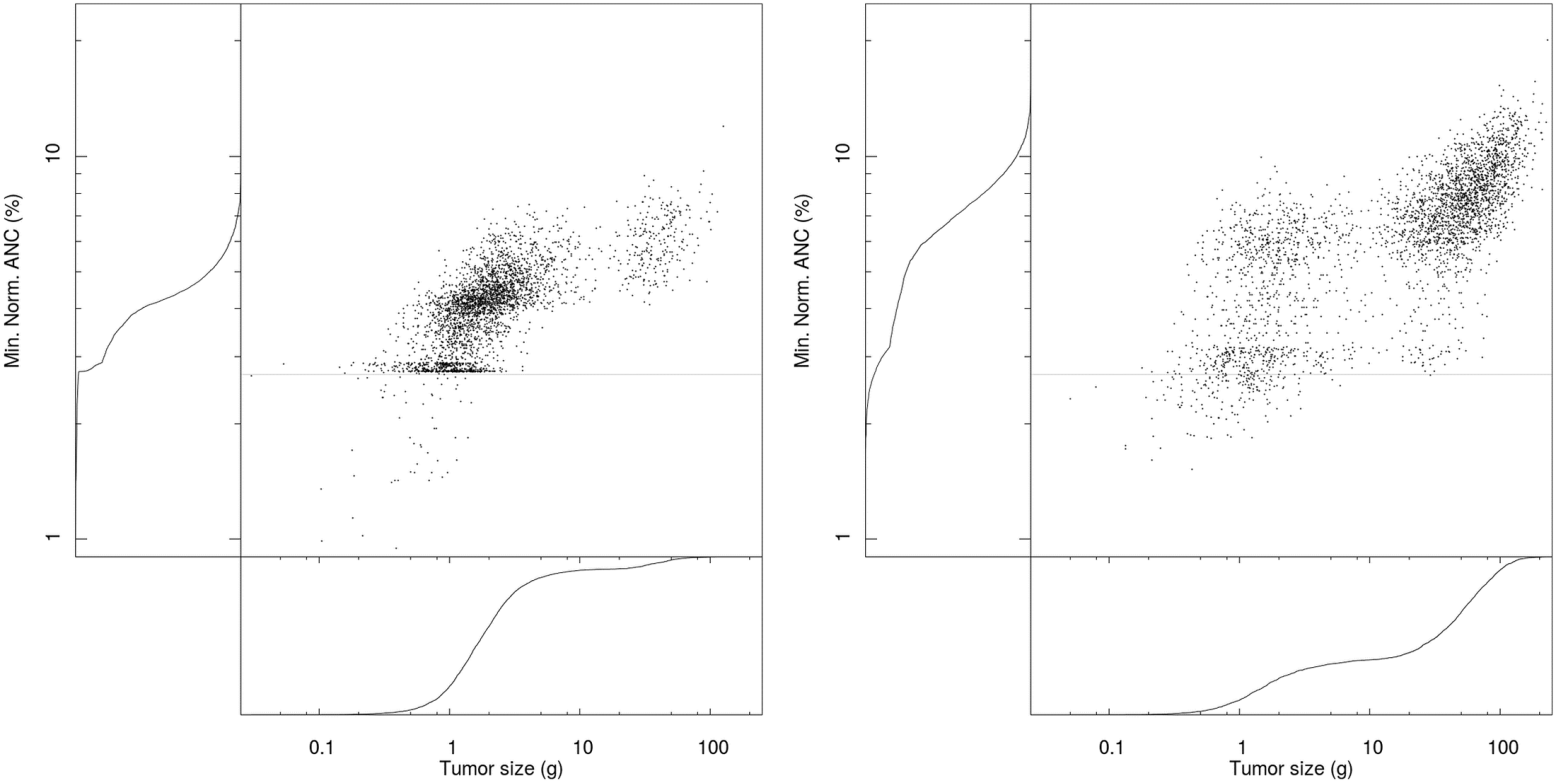
Scatter plot of protocol efficacy and toxicity for 3,200 patients. Left-hand side and bottom graphs: Cdf of toxicity and efficacy respectively. Light grey horizontal line in the central plot: 2.7% toxicity limit. Panel A: H protocol. Panel B: MTD protocol.

Elements of Table 2 are confirmed by scatter plots in Fig 2, which represent the pair (efficacy, toxicity) for each of the 3,200 patients of our sample population. Left-hand side and bottom graphs are the empirical cumulative distribution function (cdf) for toxicity and efficacy respectively. The comparison of both panels in Fig 2 makes it clear that the H protocol offers a better efficacy in terms of average and of dispersion. If this can be seen on the scatter plot, this is particularly visible on the cdf graphs for tumor sizes (bottom graph for both panels). We can for instance observe that more than 90% of patients with H protocol involve a tumor mass smaller than 10 grams, while this proportion barely amounts to 40% with MTD. This better efficacy comes from a better control of normalized ANC. Compared to MTD, a higher number of patients reach a normalized ANC nadir close to the acceptability threshold, while a smaller proportion crosses the threshold. This can be seen visible on the cdf graph for normalized ANC (left graph for both panels), where the cdf is more tilted toward the toxicity threshold with H protocol than with MTD. We also observe a sharp increase in the cdf for the H protocol right above the threshold, reflecting than few patients will experience a below-threshold normalized ANC nadir.

We can also compare more precisely the two protocols patient-wise, since populations to which the MTD and H protocols have been administered are identical. First, regarding toxicity, each patient experiencing a normalized ANC nadir below the acceptability threshold with the H protocol, also experiences a below-threshold ANC nadir with MTD. In other words, if the toxicity level for a given patient is too high with the H protocol, switching to MTD will not restore an acceptable toxicity level. Second, patient-wise efficacy comparisons are also unambiguous. For each of the 3,200 patients in the population, the H protocol yields a strictly smaller tumor size than MTD. Not only the H protocol has a better efficacy than MTD, in terms of average and of dispersion, but the former also offers a strictly better efficacy than the latter for each and every patient, with no toxicity aggravation.

**Fig 3:**
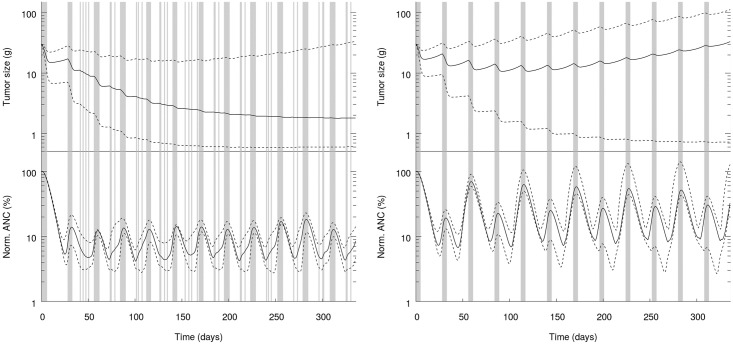
Tumor size (top) and normalized ANC (bottom) as a function of time. Grey areas: Treatment periods; solid line: Median; dashed lines: 5th and 95th percentiles. Left panel: H protocol. Right panel: MTD protocol.

Finally, we report in Fig 3 the evolution over time of the efficacy and toxicity for both the MTD and H protocols. So as to ease the comparison between MTD and H protocols we have reproduced the graph for the MTD case (panels B in Figs 1 and 3 are the same).

Fig 3 is another confirmation of the better efficacy-toxicity trade-off offered by the H protocol compared to MTD. In Fig 3, we plot the time evolution of the tumor size (top graph in each panel) and the normalized ANC (bottom graph in each panel) for a population to which either the H protocol (left panel) or the MTD protocol (right panel) has been administered. On each graph, the grey areas materialize the treatment dates, while blank areas correspond to rest days. For MTD, we can observe the cycles of 5 consecutive treatment days followed by 23 rest days. Finally, we plot three lines on each graph. The plain black line corresponds to the median value, while bottom and top dashed lines correspond to the 5th and 95th percentiles, respectively. Of note, we use a log-scale for y-axis.

We can draw several lessons from Fig 3. First, it confirms the better efficacy of the H protocol compared to MTD. The median tumor size is stabilized at a low value, while with MTD the median tumor size ends up increasing despite the treatment. The 95th percentile with H has also a better behavior than with MTD, even though we do not observe a tumor size stabilization. Second, the profiles of normalized ANC are also very different. Consistently with our toxicity constraint, the 5th percentile of normalized ANC has higher values with H than with MTD –and thereby reflecting a less severe toxicity with H than with MTD. However, the median and the 95th percentile for the H protocol feature smaller values than those for the MTD protocol. This confirms that the H protocol better manages normalized ANC, which is probably one of the reasons explaining its better efficacy.

Finally, regarding the patterns of treatment and rest periods, we can observe a pseudo-periodicity for the H protocol. This pseudo-periodicity is reminiscent of the MTD protocol cycles. Even though we do not impose any cycle, a pseudo-cycle naturally emerges in the H protocol. However, despite the resemblance with MTD, periodicity of the H protocol is not as exact as for MTD –hence, the term pseudo-periodicity. Periods of consecutive treatment days do not always exactly last 5 days and the interval between those periods does not always exactly amount to 23 days. Finally, and more substantially, the interval between the blocks of consecutive treatment days is never a full rest period but always contains a handful of treatment days (from 2 to 4). These interim treatment days seem to have a significant impact on the efficacy of the protocol, by avoiding the tumor to recover too much between treatment periods. They also influence the normalized ANC, which is, as discussed above, overall lower with H than with MTD. These interim treatment days also connect the H protocol to metronomic chemotherapy regimens, which involve low doses at a frequent schedule and without prolonged no treatment period.

### Comparison with other protocols

Since the curse of dimensionality prevents an actual optimization to be conducted in this set-up, there is no obvious protocol to which we can compare the H protocol. For this reason, we have chosen to compare the outcomes of our optimal protocol to those of a large family of protocols generalizing MTD. More precisely, we will consider the set of protocols {*P*(*x*, 28 − *x*):*x* = 1, …, 27}. A protocol *P*(*x*, 28 − *x*) consists of 12 cycles of 28 days, where each cycle starts with *x* consecutive days of treatments followed by 28 − *x* rest days. Of note, MTD can be seen as *P*(5, 23).

We report the results in Table 3 for *x* varying from 1 to 10. For the sake of convenience, we also repeat the results of the H protocol. The full results, for *x* varying from 1 to 27 can be found in S3 Appendix. We observe that all protocols with fewer treatment days than MTD have an acceptable toxicity severity (5th percentile of ANC nadir above 2.67%) but yield much larger tumor sizes. Conversely, if some protocols with a higher number of treatment days than MTD yield small tumor masses (e.g., *P*(8, 20)), this comes at the cost of a very severe toxicity. The ANC nadir is overall very low. For instance, starting from *P*(7, 21), the median –not to mention the 5th percentile– normalized ANC is below the normalized ANC threshold. We do not report them them but all protocols with more than 11 treatment days lead to slightly lighter tumor masses, but even more severe toxicity. Finally, *P*(6, 22) yields outcomes that look “close” to those of the H protocol, but tumor masses are larger and toxicity is overall more severe. Overall, the H protocol clearly yields better outcomes than any of the *P*(*x*, 28 − *x*) protocols.

**Table 3:** Comparing H protocol to the protocol family {*P*(*x*, 28 − *x*)}. Median values and in square brackets, the 5th and 95th percentiles.

Protocol	Tumor mass (g)	Norm. ANC nadir (%)
H protocol	1.80 [0.60,33.55]	4.17 [2.74,6.22]
*P*(1, 27)	301.83 [207.83,395.09]	42.33 [33.06,51.56]
*P*(2, 26)	180.65 [97.21,281.78]	22.68 [17.03,30.60]
*P*(3, 25)	113.104 [37.22,206.25]	14.17 [10.34,20.24]
*P*(4, 24)	67.7481 [1.44,153.14]	9.61 [6.69,14.43]
*P*(5, 23) (MTD)	32.99 [0.72,111.40]	6.74 [2.67,10.76]
*P*(6, 22)	2.76 [0.52,80.83]	3.54 [0.97,8.23]
*P*(7, 21)	1.56 [0.41,54.70]	1.27 [0.54,6.36]
*P*(8, 20)	1.26 [0.34,32.97]	0.71 [0.34,3.97]
*P*(9, 19)	1.11 [0.29,5.03]	0.46 [0.24,1.71]
*P*(10, 18)	1.02 [0.24,2.76]	0.32 [0.12,0.97]

## Discussion

We have proposed a novel algorithm for the optimization of temozolomide protocols, by taking into account a multiple-objective criterion. Our H protocol features a much better efficacy than the standard MTD. The efficacy, in terms of both average value and of dispersion is unambiguously in favor of the H protocol compared to MTD. This better efficacy can partly be explained by a better management of toxicity. On the one hand, a smaller share of the population experiences a toxicity below the acceptability threshold, and on the other hand, the toxicity for all patients is overall closer to the acceptability threshold. It is noteworthy that our algorithm is very flexible. In particular, the algorithm is able –with no added complexity– to handle a multidimensional non-linear objective and to address population variability.

Our article can also be seen as a first and successful step toward the introduction of methods borrowed from operational research and artificial intelligence into the realm of protocol design in oncology.

## Supporting information

S1 Appendix PK/PD model. We describe the equations for the temozolomide PK/PD model and its calibration.
(PDF)

S2 Appendix Generalization: Considering various acceptable toxicity thresholds. We present the efficacy and toxicity results optimal protocols for several calibrations of parameter *θ* in Algorithm 1.
(PDF)

S3 Appendix Robustness check. We provide the detailed results of two other algorithm calibrations, which respectively correspond to a 0% and a 7% target population share. We also provide the complete results for protocols {*P*(*x*, 28 − *x*): *x* = 1, …, 27}.
(PDF)
