# Supplementary material for: Optimal dynamic regimens with artificial intelligence: The case of temozolomide
Source: PLoS One. 2018 Jun 26;13(6):e0199076. doi: 10.1371/journal.pone.0199076 (PMC6019254; doi:10.1371/journal.pone.0199076)
Supplement: S1 Appendix — We describe the equations for the temozolomide PK/PD model and its calibration. (PDF) [file pone.0199076.s001.pdf]

PK/PD Model
S1 Appendix for "Optimal dynamic regimens with artificial intelligence"

Nicolas HOUY François LE GRAND*

Model equations

The equations of the PK/PD model we use are given below.

$$\begin{cases} \dot{y_1} &= -k_a.y_1 + u(t) \\ \dot{y_2} &= -k_e.y_2 + \frac{k_a}{V} y_1 \\ \dot{y_3} &= -a_1 \exp(-b_1.y_3)y_3 + (y_2 - c_1)H(y_2 - c_1) \\ \dot{y_4} &= -a_2 \exp(-b_2.y_4)y_4 + (y_2 - c_2)H(y_2 - c_2) \\ \dot{y_5} &= \lambda y_6 \log\left(\frac{K}{y_5}\right) y_5 - \exp(-r.y_7)u_1.y_3.y_5 \\ \dot{y_6} &= 1 - (1 + u_2.y_4)y_6 \\ \dot{y_7} &= (y_2 - c_1)H(y_2 - c_1) \\ \dot{y_8} &= \left[H(K_D - y_2).\left(r_{max} - (r_{max} - r_{min}).\left(\frac{y_{11}}{y_{11}+K_m}\right)\right) - k_1\right] y_8 \\ \dot{y_9} &= k_1.y_8 - k_2.y_9 \\ \dot{y_{10}} &= k_2.y_9 - k_3.y_{10} \\ \dot{y_{11}} &= k_3.y_{10} - k_{el}.y_{11} \end{cases} .$$

We use the following initial conditions.

$$\begin{cases} y_1(0) = y_2(0) = y_3(0) = y_4(0) = y_7(0) &= 0 \\ y_5(0) &= 30 \\ y_6(0) &= 1 \\ y_{11}(0) &= K_m\left(\frac{r_{max}-k_1}{k_1 - r_{min}}\right) \\ y_{10}(0) &= k_{el}.y_{11}(0)/k_3 \\ y_9(0) &= k_3.y_{10}(0)/k_2 \\ y_8(0) &= k_2.y_9(0)/k_1 \end{cases} .$$

H denotes the Heaviside function (*i.e.*, $H(x) = 1$ for $x \geq 0$ and 0 otherwise). Other variables have the following meaning:

*houy@gate.cnrs.fr and legrand@em-lyon.com.

- y_1, y_2: one-compartment model for the PK modelling of temozolomide;
- y_2 is the temozolomide plasmatic concentration;
- y_3 to y_7 model the PD for efficacy;
- y_3 models the effect of temozolomide on cancer cells;
- y_4 models effect of temozolomide on endothelial cells;
- y_5 is the tumor size in grams;
- y_6 models temozolomide anti-angionic effect; the effect is present when $y_6 < 1$ and absent when $y_6 = 1$;
- y_7 represents the area under the curve of plasmatic concentration y_2 with a threshold c_1;
- y_8 to y_{11} model the PD for toxicity;
- y_8 is the proliferating cell count in the bone marrow (all types combined);
- y_9 is the non-proliferating cell count in the bone marrow in early maturation stages (metamylocytes);
- y_{10} is the non-proliferating cell count in the bone marrow in later maturation stages (bands and segmented);
- y_{11} is the neutrophil count (in % of the beginning-of-treatment neutrophil count).

Model calibration

S1 Table 1 gathers the parameter values we consider.

S1 Table 1: **Parameters' values**

Parameter	Value
a_1	0.7
a_2	0.27
b_1	0.31
b_2	0.31
c_1	3.7
c_2	0.3
λ	0.00551/d

Parameter	Value
r	0.07
r_{max}	0.13/h
r_{min}	0.0445/h
k_1	0.046/h
$k_2 = k_3$	(2./9.2)/d
k_{el}	(1./6.8)/h
$K_D = K_m$	0.009

Authors of [1] have set the parameters of the left column (except λ) to physiologically plausible values that enable to reproduce some stylized facts about the MTD protocol. However, these parameters have not actually been estimated based on clinical trials. The parameters in the right column, as well as the parameter λ, have been estimated in [2] on to match the outcomes of a clinical trial.

The population pharmacokinetics parameters are gathered in S1 Table 2. All parameters follow a log-normal distribution $\mathcal{LN}(\mu, \sigma^2)$ and we report for each parameter the distribution parameters μ and σ^2 (the mean and variance of the logarithm of the parameter, respectively). We also report the mean and the standard error of the parameter. These latter quantities come from Panetta et al. [3], who have estimated population variability in temozolomide pharmacokinetics using results of a clinical trial.

S1 Table 2: **Population parameters' values**

Parameter	μ (mean of log)	σ^2 (variance of log)	Mean	Standard error
k_a (day^{-1})	4.053	10^{-4}	57.6	0.58
k_e (day^{-1})	2.218	0.036	9.36	1.80
V (L)	2.629	0.021	14.0	2.04

The population of 3,200 patients that we consider in the paper differs along the values of their pharmacokinetic parameters. We have randomly drawn these values using the log-normal distributions that are specified in S1 Table 2, assuming that patients are independent from each other and that the different parameters of a given patient are also independent from each other. Because of space constraints, we cannot report here the 9,600 parameter values that we have drawn. However, they are not mandatory for replicating our study.

Indeed, given the size of the population, any other draw of 3,200 patients would enable to very closely replicate our results.

Finally, for the simulations with no variability, we use the parameter mean values.

References

[1] Faivre C, Barbolosi D, Pasquier E, Andre N. A mathematical model for the administration of temozolomide: comparative analysis of conventional and metronomic chemotherapy regimens. Cancer Chemother Pharmacol. 2013;71(4):1013–1019.

[2] Panetta JC, Kirstein MN, Gajjar AJ, Nair G, Fouladi M, Stewart CF. A mechanistic mathematical model of temozolomide myelosuppression in children with high-grade gliomas. Math Biosci. 2003;186(1):29–41.

[3] J. C. Panetta, M. N. Kirstein, A. Gajjar, G. Nair, M. Fouladi, R. L. Heideman, M. Wilkinson, and C. F. Stewart. Population pharmacokinetics of temozolomide and metabolites in infants and children with primary central nervous system tumors. *Cancer Chemother. Pharmacol.*, 52(6):435–441, Dec 2003.
