# Supplementary material for: Optimal dynamic regimens with artificial intelligence: The case of temozolomide
Source: PLoS One. 2018 Jun 26;13(6):e0199076. doi: 10.1371/journal.pone.0199076 (PMC6019254; doi:10.1371/journal.pone.0199076)
Supplement: S2 Appendix — We present the efficacy and toxicity results optimal protocols for several calibrations of parameter θ in Algorithm 1. (PDF) [file pone.0199076.s002.pdf]

Generalization: Considering various acceptable toxicity thresholds
S2 Appendix for "Optimal dynamic regimens with artificial intelligence"

Nicolas Houy François Le Grand*

In the main text, we assume that in the optimization algorithm, less than 2% of the population should cross the acceptable ANC nadir threshold equal to $nadir_{\min} = 2.7\%$. This population share limit of 2% is represented by the parameter θ in our algorithm. In S2 Fig 1, we plot the results for different values (from 0 to 7%) for the population share θ. Each population share is represented by a "cross", whose horizontal bar represents the 90% confidence interval for toxicity and the vertical bar, the 90% confidence interval for efficacy. The center of the cross is the median value for both toxicity and efficacy, while the two ends of the cross are the 5th and 95th percentiles. For toxicity, we report the rejection rate (in %), which corresponds to the share of the 3,200 patient population experiencing an ANC nadir below 2.7%. For efficacy, we report the efficacy gain compared to MTD. The efficacy gain is measured by the difference in decimal logarithm of tumor sizes. For instance, a value of 0.88 (corresponding to the bullet in S2 Fig 1, which materializes our benchmark H protocol) corresponds to a median difference in log of tumor sizes of 0.88. This means that tumor sizes are –on "median"– $10^{0.88} \approx 7.6$ times smaller with H protocol than with MTD.

In S2 Fig 1, the bullet corresponds to our benchmark calibration, while the circle corresponds to a target population share of 0% and the square a target population share of 7%. These calibrations are presented in greater detail in S3 Appendix.

*houy@gate.cnrs.fr and legrand@em-lyon.com.

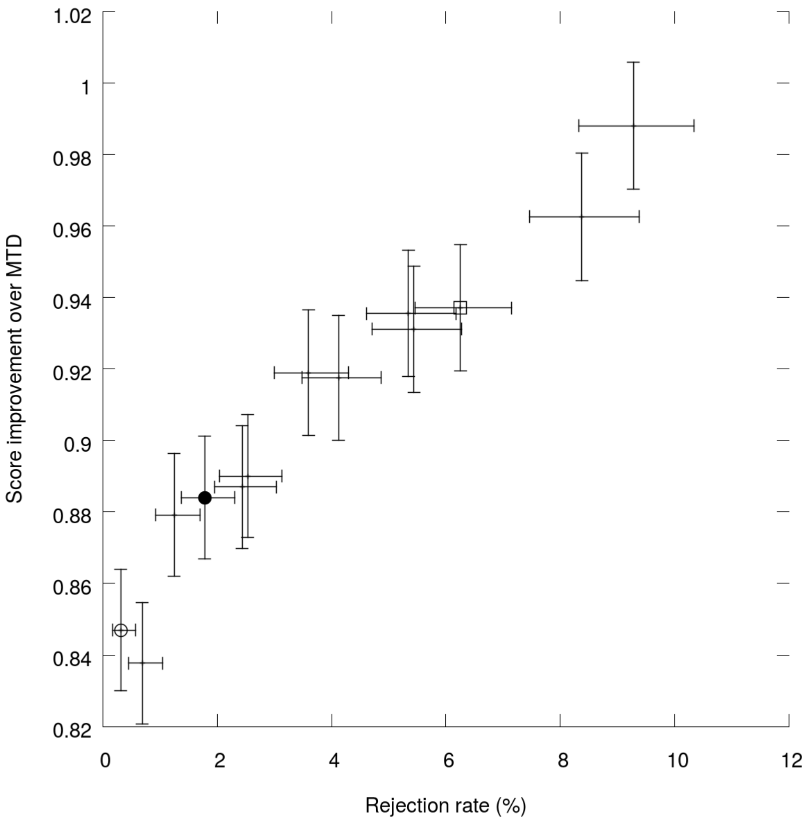

S2 Figure 1: **Distribution of efficacy and toxicity results for several algorithm calibrations.**
Bullet: benchmark calibration; circle: target population share of 0%; square: target population share of 7%.
