# Supplementary material for: Optimal dynamic regimens with artificial intelligence: The case of temozolomide
Source: PLoS One. 2018 Jun 26;13(6):e0199076. doi: 10.1371/journal.pone.0199076 (PMC6019254; doi:10.1371/journal.pone.0199076)
Supplement: S3 Appendix — We provide the detailed results of two other algorithm calibrations, which respectively correspond to a 0% and a 7% target population share. We also provide the complete results for protocols {P(x, 28 − x): x = 1, …, 27}. (PDF) [file pone.0199076.s003.pdf]

Robustness check
S3 Appendix for "Optimal dynamic regimens with artificial intelligence"

Nicolas HOUY François LE GRAND*

Two other calibrations

We present here the detailed results of two other algorithm calibrations, which respectively correspond to a 0% and a 7% target population share. We will refer to these two protocols as the 0% and 7% protocols.

We represent scatter plots in S3 Fig 1 –similar to Fig 2 in the main text. Without surprise, toxicity is more concentrated around the toxicity limit for the 7% protocol, and its efficacy is slightly better than the one of the 0% protocol. Conversely, a much smaller number of patients experience a below threshold toxicity with the 0% protocol.

Finally, we plot in S3 Fig 2 the evolution over time of the efficacy and toxicity for both the MTD and H protocols. This figure is similar to Fig 3 in the main text.

Conclusions we can draw from S3 Fig 2 are consistent with what we could have expected. Overall, the better efficacy of the 7% protocol –especially in terms of dispersion– comes from an overall more severe toxicity. Furthermore, if both protocols exhibit a pseudo-cycle as the H protocol, the main difference between both protocols lie in the interim treatments occurring between major treatment blocks. They are more frequent with the 7% protocol than with the 0% one. These results confirm our findings of the main paper and illustrate that the sensitivity of our algorithm to a key parameter is consistent with intuition.

*houy@gate.cnrs.fr and legrand@em-lyon.com.

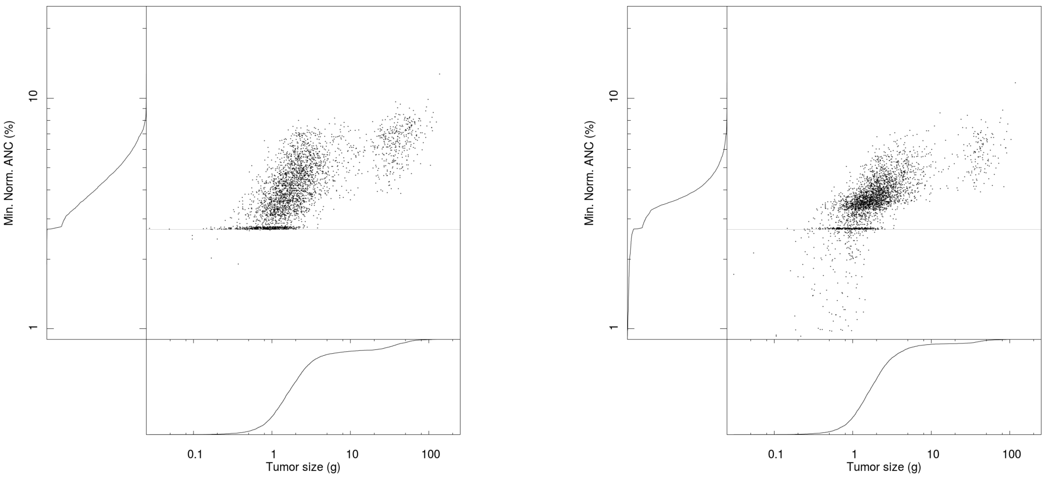

(a) 0% protocol

(b) 7% protocol

S3 Figure 1: Scatter plot of protocol efficacy and toxicity for 3,200 patients. Left-hand side and bottom graphs: cdf of toxicity and efficacy respectively. Light grey horizontal line in the central plot: 2.7% toxicity limit.

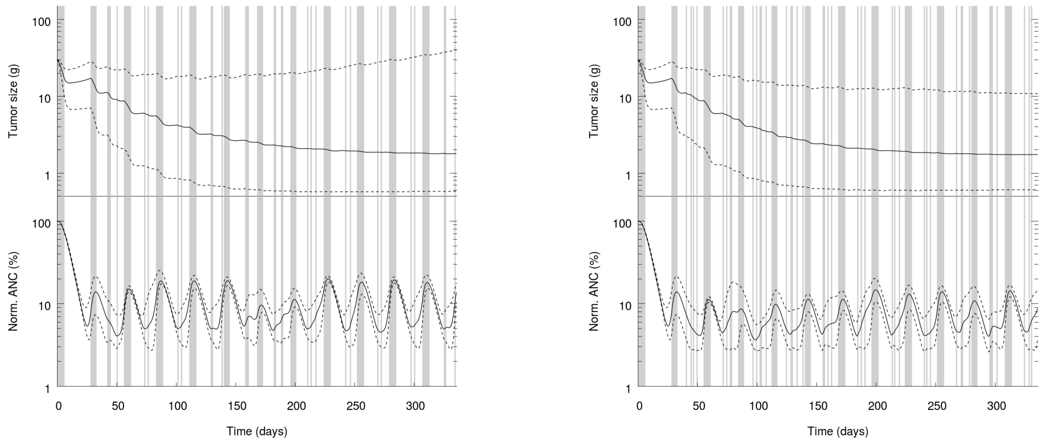

(a) 0% protocol

(b) 7% protocol

S3 Figure 2: Tumor size (top) and normalized ANC (bottom) as a function of time.
Grey areas: treatment periods; solid line: median; dashed lines: 5th and 95th percentiles.

Complete results for the protocols $\{P(x, 28 - x) : x = 1, \ldots, 27\}$

We provide here the complete results for $\{P(x, 28 - x) : x = 1, \ldots, 27\}$.

S3 Table 1: **Comparing H protocol to the protocol family $\{P(x, 28-x)\}$. Median values and in square brackets, the 5th and 95th percentiles.**

Protocol	Tumor mass (g)	Norm. ANC nadir (%)
H protocol	1.80 [0.60,33.55]	4.17 [2.74,6.22]
$P(1,27)$	301.83 [207.83,395.09]	42.33 [33.06,51.56]
$P(2,26)$	180.65 [97.21,281.78]	22.68 [17.03,30.60]
$P(3,25)$	113.104 [37.22,206.25]	14.17 [10.34,20.24]
$P(4,24)$	67.7481 [1.44,153.14]	9.61 [6.69,14.43]
$P(5,23)$ (MTD)	32.99 [0.72,111.40]	6.74 [2.67,10.76]
$P(6,22)$	2.76 [0.52,80.83]	3.54 [0.97,8.23]
$P(7,21)$	1.56 [0.41,54.70]	1.27 [0.54,6.36]
$P(8,20)$	1.26 [0.34,32.97]	0.71 [0.34,3.97]
$P(9,19)$	1.11 [0.29,5.03]	0.46 [0.24,1.71]
$P(10,18)$	1.02 [0.24,2.76]	0.32 [0.12,0.97]
$P(11,17)$	0.96 [0.21,2.22]	0.24 [0.07,0.64]
$P(12,16)$	0.91 [0.18,1.95]	0.14 [0.05,0.46]
$P(13,15)$	0.88 [0.16,1.79]	0.08 [0.03,0.33]
$P(14,14)$	0.85 [0.14,1.68]	0.06 [0.02,0.26]
$P(15,13)$	0.83 [0.12,1.59]	0.04 [0.01,0.20]
$P(16,12)$	0.81 [0.10,1.53]	0.03 [$1.53\,10^{-3}$,0.16]
$P(17,11)$	0.80 [0.09,1.48]	0.02 [$6.07e{-}05$,0.11]
$P(18,10)$	0.78 [0.08,1.44]	0.01 [$4.31e{-}08$,0.08]
$P(19,9)$	0.77 [0.06,1.41]	$4.71\,10^{-3}$ [$3.01e{-}22$,0.05]
$P(20,8)$	0.76 [0.05,1.39]	$1.59\,10^{-4}$ [$1.82e{-}28$,0.04]
$P(21,7)$	0.75 [0.04,1.36]	$2.65e-05$ [$1.82e{-}28$,0.03]
$P(22,6)$	0.75 [0.01,1.35]	$4.00e-08$ [$1.82e{-}28$,0.02]
$P(23,5)$	0.74 [$2.57\,10^{-3}$,1.33]	$8.25e-20$ [$1.82e{-}28$,0.02]
$P(24,4)$	0.73 [$6.32\,10^{-4}$,1.32]	$1.89e-28$ [$1.82e{-}28$,0.01]
$P(25,3)$	0.72 [$2.74\,10^{-4}$,1.31]	$1.89e-28$ [$1.82e{-}28$,0.01]
$P(26,2)$	0.71 [$1.98\,10^{-4}$,1.29]	$1.89e-28$ [$1.82e{-}28$,$3.53\,10^{-3}$]
$P(27,1)$	0.70 [$1.91\,10^{-4}$,1.28]	$1.89e-28$ [$1.82e{-}28$,$5.74\,10^{-4}$]
